# Recent Developments in ZnS-Based Nanostructures Photocatalysts for Wastewater Treatment

**DOI:** 10.3390/ijms232415668

**Published:** 2022-12-10

**Authors:** Luminita Isac, Alexandru Enesca

**Affiliations:** 1 Product Design, Mechatronics, and Environmental Department, Transilvania University of Brasov, 500036 Brasov, Romania; 2 Renewable Energy Systems and Recycling Research Center, Transilvania University of Brasov, 500036 Brasov, Romania

**Keywords:** ZnS nanostructures, heterojunctions, photocatalysis, organic pollutants, wastewater treatment

## Abstract

The continuous growth of the world population has led to the constant increase of environmental pollution, with serious consequences for human health. Toxic, non-biodegradable, and recalcitrant organic pollutants (e.g., dyes, pharmaceuticals, pesticides) are discharged into water resources from various industries, such as textiles, leather, pharmaceuticals, plastics, etc. Consequently, the treatment of industrial wastewater, via a sustainable technology, represents a great challenge for worldwide research. Photocatalytic technology, an innovative technique based on advanced oxidation process (AOP), is considered a green technology with promising prospects in the remediation of global environmental issues. In photocatalysis, a very important role is attributed to the photocatalyst, usually a semiconductor material with high solar light absorption capacity and conductivity for photogenerated-charge carriers. Zinc sulfide (ZnS), as n-type semiconductor with different morphologies and band gap energies (Eg = 3.2–3.71 eV), is recognized as a promising photocatalyst for the removal of organic pollutants from wastewater, especially under UV light irradiation. This review deals with the recent developments (the last five years) in ZnS nanostructures (0D, 1D, 3D) and ZnS-based heterojunctions (n-n, n-p, Z scheme) used as photocatalysts for organic pollutants’ degradation under simulated (UV, Vis) and sunlight irradiation in wastewater treatment. The effects of different synthesis parameters (precursors’ type and concentration, capping agents’ dosages, reaction time and temperature, metal doping, ZnS concentration in heterostructures, etc.) and properties (particle size, morphology, band gap energy, and surface properties) on the photocatalytic performance of ZnS-based photocatalysts for various organic pollutants’ degradation are extensively discussed.

## 1. Introduction

Wastewater loaded with toxic, non-biodegradable organic pollutants represents a real problem, both for aquatic environments and for human life and development. Industrial wastewater originates mainly from the textile industry, but also from the plastics, paper, food, and cosmetic industries, due to the large amount of water used in the various technological stages, especially during the dyeing process [1]. It has been estimated that the wastewater from textile production contributes approximately 20% of the total industrial polluted water [2].

The presence of a very small concentration of organic dyes in water (less than 1 ppm for some dyes) may have serious consequences for the environment, but more for the population health, such as skin and eyes irritations, respiratory and gastric tracts affections, quadriplegia, tachycardia, tissue necrosis, and cancer [1,2,3,4]. 

The dyes most used industrially are azo dyes (usually with an aromatic structure) which, under natural conditions, require a long time to degrade due to their high photostability [5]. In addition, wastewater with complex composition and high concentration in dyes is difficult to biodegrade; therefore, it is necessary to remove dyes from wastewater before discharge [6].

The worldwide increase in pharmaceuticals consumption, especially antibiotics, produces a large amount of residue detected in surface water, soil, sewage, and wastewater from pharmaceutical industry. Prolonged accumulation of pharmaceutical contaminants in water and ecosystems can lead to increased resistance of pathogens and different strains of microorganisms to antibiotics and other drugs, causing diseases that require new antibiotics/drugs production [7,8]. As a consequence, the removal of pharmaceutical products, along with other organic pollutants (dyes, herbicides) from wastewater, is considered an important target in environmental remediation. 

Although various wastewater treatment processes are currently used, the selection of an effective technological and commercial technique still remains a great challenge. Many technologies, such as physical (screening, sedimentation, aeration, adsorption, reverse osmosis, etc.), biological (biofiltration, biological nitrification–denitrification, bioremediation, aerobic/anaerobic, biosorption, membrane bioreactor—MBR, microbial fuel cell, etc.), and chemical (ion exchange, electrolysis, ozonation, photolysis, sonolysis, photo-Fenton, and chemical, photo-, electro-, and photoelectrocatalysis) have been developed for removing organic pollutants from wastewater [1,2,7,9,10]. Currently, since individual techniques do not effectively remove pollutants from wastewater, hybrid technologies have been developed that are sustainable, stable, and more efficient, both in terms of pollutant degradation and in energy-saving. Accordingly, hybrid techniques, e.g., combined ozonation with activated carbon or anaerobic photocatalysis combined with membrane processes, have shown to be significantly effective in the degradation of certain harmful organic pollutants, especially pharmaceuticals and pesticides, from wastewater [11,12].

Among chemical treatment technologies, the advanced oxidation processes (AOP) have emerged as a new, effective technique based on extremely reactive oxidant species (hydroxyl and superoxide ion free radicals) production, which decompose organic pollutant molecules into harmless and controllable by-products (e.g., H_2_O, CO_2_) [10]. 

Photocatalysis, an AOP using semiconductor materials as catalysts [13], has attracted considerable attention as a sustainable, eco-friendly, and highly efficient technology with promising prospects in global environmental issues remediation. The semiconductors-based photocatalysis has been demonstrated to be a simple, ecological, low-cost, and very efficient method for wastewater treatment [14,15]. In this technology, the semiconductor material is activated by light (natural, artificial) with energy higher than its band gap to promote the formation of energy-rich electron–hole pairs that can be involved in photocatalytic (reduction and oxidation) processes. Thus, the semiconductor photocatalyst selection must be carefully done to accomplish certain requirements: suitable band gap energy for light absorption, photochemically and photocorrosion stability, non-toxicity, and low-cost. 

As an important class of semiconductor photocatalysts, metal sulfides nanostructures attracted much attention in the past years due to their special physical and chemical properties, mainly related to the band gap energy which can be easily tuned by tailoring the particles size and morphology, without changing the chemical composition of the metal sulfide [16,17].

In the extremely varied family of transition metal semiconductors, zinc sulfide (ZnS) is a n-type semiconductor with wide band gap (2.6–4.6 eV), high electronic mobility, good thermal stability, non-toxicity, water insolubility, and quite low cost [18]. According to the literature [19,20,21,22], ZnS nanostructures (ZnS NSs) are efficient photocatalysts for organic pollutants degradation under UV light irradiation, but less active in Vis and sunlight, due to the wide band gap energies and intrinsic defects, resulting in a low number of photogenerated carriers, poor charge carriers mobility, and rapid photogenerated species recombination. These drawbacks somewhat limit the practical applications of ZnS as a photocatalyst. To overcome these impediments, various strategies have been used to improve the photocatalytic performance of ZnS NSs by adjusting band gap energy (to absorb Vis light), such as morphology engineering [23,24], metal/non-metal doping [3,9,25,26], defect engineering [26,27,28,29], dye sensitization [18], and heterostructures construction [30,31,32,33,34,35,36]. However, nanostructured ZnS materials have versatile potential applications in optoelectronic devices (e.g., flat-panel displays, injection lasers, UV light-emitting diodes, electroluminescent sensors, and infrared windows [5,37,38]) and as photocatalysts in the following processes: (a) green synthesis of organic compounds (e.g., substituted tetrazoles and xanthene and its derivatives [5]), (b) syngas production in water [39], (c) CO_2_ photoreduction [40], and (d) H_2_ production via water splitting [27,41,42,43].

The photocatalytic production of hydrogen from water has been intensively studied in recent decades as the main contribution to solve both environmental and energy issues in the future. In this context, the development of metal (Zn) sulfide-based photocatalysts for hydrogen evolution as green energy was the topic of a few recent state-of-the-art review papers [18,43].

To date, various ZnS-based photocatalysts with applications in environmental pollution, especially in wastewater treatment, were reported. This review is an up-gradation of the literature information from the last five years related to the development of ZnS-based photocatalysts used for degradation of different organic pollutants (e.g., organic dyes, pharmaceutical active compounds, phenol and phenolic compounds) under simulated (UV, Vis) and sunlight irradiation. The study aims to identify the photocatalytic performances of ZnS-based photocatalysts in the degradation of industrial organic pollutants, for possible future improvements in their efficiency in wastewater treatment. 

## 2. ZnS Nanostructures Photocatalysts for Organic Pollutants Degradation

Zinc sulfide (ZnS), an important II–VI class semiconductor photocatalyst, has two crystalline phases, sphalerite (blende, cubic lattice, space group F-43m) and wurtzite (hexagonal lattice, space group P63mc). The structures consist of ZnS4 and SZn4 tetrahedra, respectively, which occupy similar positions in the crystal lattice, but at different distances and angles. ZnS sphalerite (blende) has four asymmetric units in its cubic unit cell, while wurtzite unit cell contains two asymmetric units. The two crystalline phases of ZnS are polymorphs, the transition of ZnS blende (stable at room temperature) to ZnS wurtzite occurring in time, at ~1020 °C [18,31].

**Figure 1:**
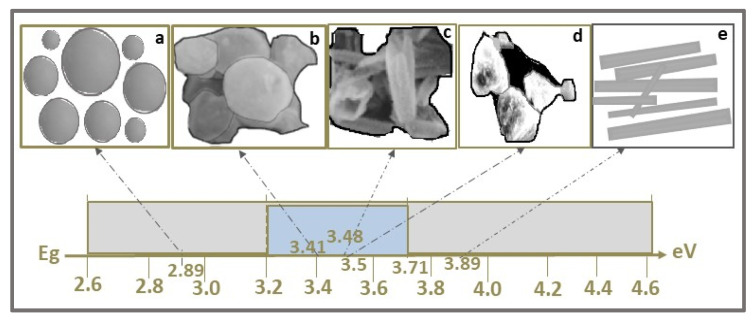
ZnS band gap energy values and their variation with ZnS NPs’ morphologies: spheres (**a**), nanosheets (**b**), nanotubes (**c**), random (**d**), and rod-shape (**e**).

ZnS is a wide band gap semiconductor, with band gap energy in the range 3.25–3.74 eV [24,44] for cubic and 3.52–3.64 [28,45] for hexagonal structure, and with high optical transmittances in the Vis region and large exciton binding energy (40 meV) [46]. The band gap energy (E_g_) reported for ZnS covers a wide range of values, from 2.5 eV [47] to 4.64 eV [48], most with Eg = 3.21–3.71 eV, as is shown in Figure 1. Thus, ZnS is more responsive to the absorption of UV light (λ < 340 nm), which represents approximately 4% of the total amount of sunlight. To extend the applications of ZnS photocatalysts in Vis light (λ ≥ 420 nm) of the solar spectrum by narrowing the band gap energy and adjusting CB/CV band-edge positions, many research groups have attempted different strategies, such as morphology variation [24,38,49], metal/nonmetal ion doping [50,51], semiconductors nanocomposites developing [52], and surface defects controlling [27]. 

It is well known that the photocatalytic activity of a semiconductor material significantly depends on its crystalline structure (particles sizes and shapes), surface morphologies, defects, etc., and ZnS nanostructures are expected to exhibit enhanced photocatalytic activity compared with bulk ZnS [53].

Depending on morphology, ZnS NSs can be classified into four groups: 0D (QDs), 1D (nanospheres, nanotubes, nanorods, nanowires, etc.), 2D (nanoplates, nanofilms, 1D nanostructure arrays, etc.), and 3D (hierarchical assemblies of 1D/2D NSs) [26,37]. The morphology strongly influences the ZnS nanostructures’ photocatalytic performance, due to the direct effect on the distribution of photogenerated electron-hole pairs and the ability to absorb reactants. Consequently, 1D NSs are suitable to the photogenerated electrons and holes transport, collection, and distribution, while 3D NSs, with a large surface area and high porosity, could provide more surface-active sites and facilitate the reactants’ and products’ transport [38].

The photocorrosion stability of ZnS and ZnS-based nanostructures must be considered also in the conditions where the organic pollutants photodegradation occurs in a water environment. Although ZnS is stable in water, in the absence of a sacrificial agent (e.g., organic or inorganic capping agent), the corrosion of ZnS-based photocatalysts could be induced by the oxidation reactions of active sites at the crystal surface by photogenerated holes [18]. The final products of the corrosion process are Zn^2+^ and elemental sulfur, contributing to metal ions’ leaching and the photocatalyst contamination with S particles. In absence of light, the ZnS NPs may also dissolve in water, due to the oxidation of S^2-^ ions from dissolved oxygen. For a better understanding of CdSe/ZnS-MPA-capped QDs’ reactivity in aphotic water environments, Paydary P. and Larese-Casanova P. [54] investigated the chemical factors that control the metal sulfides’ dissolution. The results showed that at neutral pH and in the absence of dissolved oxygen, strong chelating agents, and oxidants (H_2_O_2_), the ZnS QDs were progressively dissolved within weeks. The photochemical stability of metal sulfide (ZnS)-based photocatalysts can be improved both by controlling chemical parameters related to the preparation method and/or the pollutant solution and by designing and synthesizing ZnS nanocomposites including a cocatalyst (noble metal, metallic oxides and/or sulfides, etc.).

In general, ZnS photocatalysts’ properties can be tailored by selecting a simple, fast, ecological, and low-cost synthesis method, with convenient adjusting conditions (working parameters). 

In addition, in the photodegradation process, other important factors have to be considered, such as the photocatalyst amount, the type and concentration of pollutant, and the type and intensity of irradiation source. 

A summary of recent studies on ZnS-based nanostructures, obtained by various methods, and their photocatalytic performances in degradation of organic pollutants, using different light sources, is presented in Table 1.

### 2.1. 0D ZnS-Based Nanostructures

Zero-dimensional (0D) nanostructures (e.g., quantum dots, nanodots, magnetic nanoparticles, fullerene, polymer dots) have all three dimensions at nanoscale, consisting mostly of spherical or quasi-spherical nanoparticles with a diameter < 100 nm. Due to the structural properties, such as ultra-small dimensions and high specific areas, 0D nanostructured materials have more active sites per unit mass and quantum confinement effects within a certain dimension [60].

As 0D nanostructured materials with excellent optical and electronic properties (nontoxicity, physico-chemical stability, cost-effective synthesize procedures, etc.), ZnS QDs have received increased attention in recent years as promising photocatalysts for organic pollutants’ removal from wastewater. However, the wide band gap energy (in the range 3.7 [3]–4.64 eV [48]), the high agglomeration of small-size particles in colloidal system, and the hydrophobicity limit ZnS QDs’ photocatalytic performance, especially under Vis light irradiation. For example, nanostructured ZnS catalyst, crystallized in cubic phase and containing spherical NPs (QDs) with an average size of 4.2 nm, was prepared in a basic aqueous medium at mild temperatures (60–80 °C) using metal xanthate (potassium ethylxanthate) as a sulfur precursor without an addition of any organic solvent or surfactant [55]. The photocatalytic tests were performed in visible light (1000 W tungsten-halogen lamp) for a degradation of 100 mL MB solution (8 × 10^−6^ mol/L) by ZnS catalyst (10 mg). After 180 min of irradiation, the MB dye degradation was 61% (Table 2), which was somewhat expected, considering the large band gap energy (4 eV) due to the quantum confinement effect of ZnS QDs and, therefore, the ability to absorb more efficiently UV light.

To provide colloidal stability to ZnS nanoparticles in pollutant solution, organic capping agents, such as polymers (e.g., cellulose, chitosan [6], maleic anhydride—MA/octene-1 copolymer [61]), were used in the preparation procedure. The active parts of the polymer can be adsorbed on the ZnS NP surface, passivating it and thus ensuring thermal stability, reduced reactivity, and a controlled growth that prevent any further aggregation to particles [54,61].

To overcome the limitation related to wide band gap energy of ZnS QDs, one of the strategies frequently used by researchers is the doping of ZnS QDs with appropriate elements (e.g., metal atoms or ions), thus favoring electronic transitions at energies corresponding to Vis light wavelengths. Dopants in the photocatalyst act not only as Vis light absorption centers but also as charge carriers’ (electrons or holes) trapping sites, which avoid recombination and promote charge separation required for the photocatalytic reactions [3,18]. 

The effects of doping metals (Mn, Ag, Au, Gd) on ZnS QDs band gap energies and surface charges and, therefore, on the 0D ZnS nanostructures’ photocatalytic activity, still remain a challenging topic for many research groups [3,48,51].

**Figure 2:**
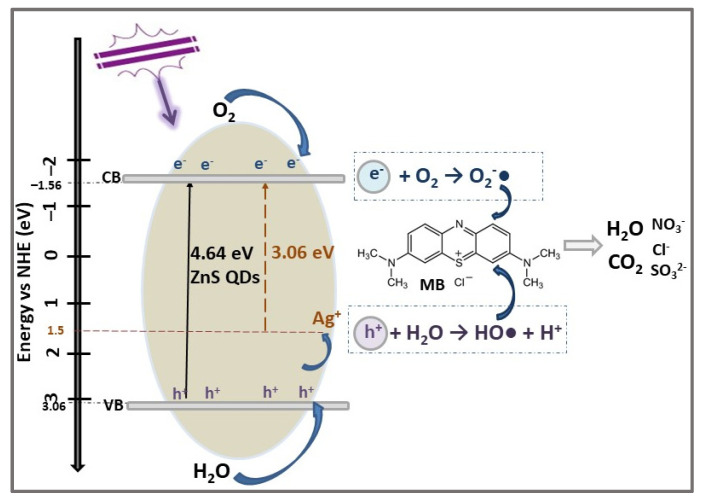
The photocatalytic degradation mechanism of MB dye using Ag-doped ZnS QDs photocatalyst under UV light irradiation for 3 h.

Madkour and Al Sagheer [48] developed a facile, room temperature, one-pot, aqueous-solution-based method for the synthesis of dispersed, undoped ZnS QDs and Ag (Au)-doped ZnS QDs. The method used a PVP/PVA polymer blend as growth media and a noble metal amount in ZnS QDs of about 4% (weight). The ZnS QDs, with particle size of 4.3 nm and Eg = 4.64 eV, degraded 72.5% MB dye (30 mg/L) under UV light irradiation after 180 min. The MB photodegradation efficiencies for doped Ag-ZnS QDs and Au-ZnS QDs increased to 92.6%, and 96.5%, respectively, in the same irradiation conditions. The enhanced photocatalytic efficiencies of Ag/Au-doped ZnS QDs are explained on the basis of the Schottky barrier of noble metal, which prevents the recombination of charge carriers, thus extending the photogenerated electrons’ lifetime [48]. The structural, optical, and photocatalytic properties of undoped and doped ZnS QDs are summarized in Table 2. The photodegradation mechanism of MB by Ag-ZnS QDs is schematically presented in Figure 2. Under UV radiation, the photo-induced electron-hole pairs were generated on the VB and CB and transferred to the ZnS QDs surface. By doping with Ag^+^ ions, a donor level above the original VB is obtained to improve the photocatalyst response in UV light by promoting photogenerated holes from VB of ZnS to Ag level. These holes react with water and form hydroxyl radicals (HO•), while photogenerated electrons from ZnS QDs CB generate the reduction reaction of O_2_ molecules to anionic superoxide radicals (O_2_^−^•). Both radicals act as strong oxidants for degradation and mineralization of MB dye molecules and transform them into CO_2_, H_2_O, and degraded products [44,50].

Investigations on ZnS QDs-based photocatalyst regeneration showed that it can be easily reactivated after five cycles of photodegradation, using a facile washing procedure. After reactivation (sixth cycle), the recovery efficiency became 92.5% for ZnS QDs, 89.6% for Ag-ZnS QDs, and 90% for Au-ZnS QDs, compared to the first cycle photodegradation efficiency [48].

The ZnS QDs and Gd-doped ZnS QDs (2 wt%) were recently synthesized by refluxing the precursors’ solutions (containing Cu^2+^, S^2−^, and Gd^3+^) for 3 h at 100 °C in a nitrogen-rich atmosphere [51]. The structural, optical, and photocatalytic properties of ZnS QDs and Gd-doped ZnS QDs are presented in Table 2. 

The insertion of Gd^2+^ into the crystalline lattice of the ZnS QDs had, as a result, an increase of photocatalytic activity in degradation of dye AR14 under UV illumination for 180 min. The enhanced photocatalytic activity of Gd-ZnS QDs can be attributed to the efficient separation of electron-hole pairs in the presence of Gd^2+^ dopant. Moreover, due to the ZnS QDs’ doping with Gd^2+^ ions, the band gap energy decreased, from 3.7 eV to 3.45 eV, and the activation of the photocatalyst under UV irradiation was accelerated, thus increasing the degradation efficiency [51].

Photodegradation studies of organic dye TO under UV irradiation in presence of pure and Mn-doped ZnS QDs, prepared by a simple reflux method, were recently reported by Velázquez et al. [3]. The photodegradation of TO was found to be dependent on the ZnS QDs’ concentration (250 ppm, 500 ppm) and exposure time in UV light (8 W lamp, 10 mW/cm^2^). Therefore, both the ZnS QD and the Mn-ZnS QD photocatalysts were more efficient when higher concentrations (500 ppm) and prolonged illumination time (90 min) were used. Due to the presence of a small amount of Mn in Mn-ZnS QDS (about 0.22 at. %, according to EDS analysis), significant differences in the structural, optical, and photocatalytic properties of the two ZnS QD photocatalysts were not identified (Table 2).

**Figure 3:**
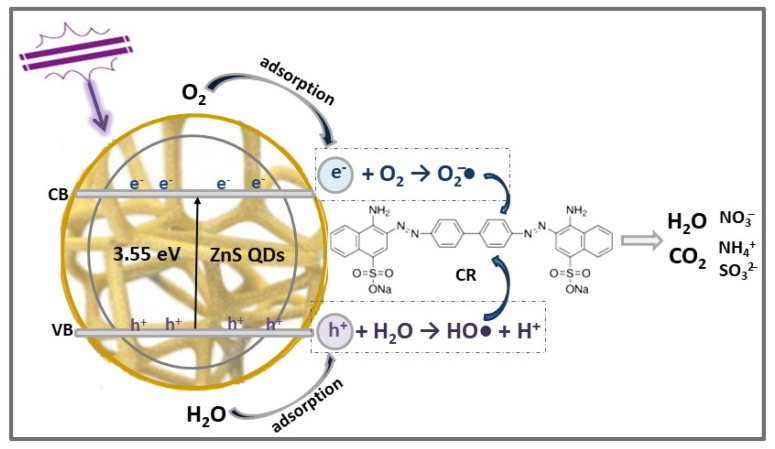
The photocatalytic degradation mechanism of CR dye using ZnCCSs’ photocatalyst under UV light irradiation.

An efficient and sustainable approach for removing organic dyes from wastewater through the combination of adsorption and photocatalytic degradation processes, using ZnS QDs immobilized in porous cellulose/chitosan sponge skeleton (ZnCCSs), was developed recently by You et al. [6]. The ZnCCSs’ photocatalyst was obtained via hydrothermal decomposition of a zinc salt solution in chitosan xanthate (ChX) and cellulose xanthate (CeX), as sulfur precursors, using PVP (2, 4, 6 wt%) as capping agent and stabilizer. The natural polymers’ cellulose and chitosan were selected as supports for ZnS QDs’ immobilization due to their special properties, such as high natural abundance, high hydrophilicity, flexibility, permeability, and non-toxicity. Moreover, these biopolymers have application in wastewater purification as biodegradable sorbents for the removal of heavy metals and various anionic dyes [62,63]. As a result of biopolymers’ and ZnS QDs’ complementary properties, ZnCCSs demonstrated high porosity (83.46%), low bulk density (28.89 mg/cm^3^), and uniformly distributed cubic ZnS NPs (15 nm size) on the sponge surface. The band gap energies, in the range 3.2–3.55 eV, depend on the PVP concentration (the Eg increases with the PVP amount increase) used in the preparation procedure and are higher than that of ZnS QDs (3.26 eV) due to the quantum size’s effect. Accordingly, the efficiency of ZnCCSs photocatalyst in CR dye solution (50 mg/L) degradation under UV light (350 W mercury lamp) irradiation for 120 min, Table 2, was also influenced by the PVP amount. Thus, when increasing PVP concentration from 2 wt% to 6 wt%, the CR removal efficiency increased from 84.96% to 95.94%, the PVP addition preventing the excessive growth and partial agglomeration, and this favors the uniform distribution of ZnS QDs on the cellulose/chitosan sponge. In the CR’s photodegradation mechanism, Figure 3, the polysaccharides’ substrates, exhibiting strong absorption ability, also provide a large number of OH^–^ ions on its surface, increasing the amount of HO• radicals resulted from reaction with photogenerated holes.

For further application on an industrial scale, the reusability of ZnCCS adsorbent and photocatalyst was investigated. The results showed very good stability after eight reusability cycles, both in adsorption and photocatalytic activities, for ZnCCS obtained under optimal conditions (cellulose:chitosan = 4:6, 3 wt% TSH, and 6 wt% PVP). The reported slight decrease of 7% was attributed to the weak UV radiation inside the sponge. A solution proposed to remedy this inconvenience could be the use of smaller pieces of sponge [6].

### 2.2. 1D ZnS-Based Nanostructures

One-dimensional (1D) nanostructures (nanospheres, nanorods, nanotubes, nanowires, etc.) have at least one dimension larger than nanoscales, and the other two dimensions are within nano range (d = 1–100 nm). Over the last decades, 1D NSs have gained increased attention due to their diversity in morphology, chemical composition, and structure. Accordingly, considerable effort has been devoted to the preparation of ZnS’ nanostructured photocatalysts with controlled morphologies, such as nanospheres [24,44,49], nanosheets, nanotubes [38], and nanorods [49]. Among 1D ZnS NSs, nanotubes have a hollow mesoporous structure, and consequently a large specific surface area, enhancing their optoelectronic properties (e.g., Eg, electron conductivity). Nanorods have solid structures, with lengths between 10 and 20 nm and usually hexagonal cross-sections, the core-shell nanorods structures being the most studied [26,64]. Various morphologies of ZnS NSs and their corresponding band gap energy values are illustrated in Figure 1.

The effect of sulfur precursors on the formation and morphological, optoelectronic, and photocatalytic properties of ZnS NSs were investigated by Ma et al. [24]. ZnS NSs with different morphologies were prepared by using a simple and capping-free HT method, with thiourea (TU), TAA, and Na_2_S as sulfur sources. While nanospheres and hierarchical spherical structures ZnS (1D NSs) were obtained with TU and TAA, bulk ZnS was formed with the Na_2_S precursor. All structures are cubic blende ZnS phase with crystallite sizes increasing from 4.58 nm (ZnS obtained with TU) to 13.1 nm (bulk ZnS), as a result of the increased releasing rate of sulfur sources. Associated with an internal stress increase, the band gap energy decreases for ZnS 1D NSs, e.g., Eg = 3.25 eV for ZnS obtained with TAA compared with bulk ZnS with Eg = 3.37 eV. The photocatalytic performances of ZnS NSs were evaluated in the degradation of MB dye solution (12 mg/L) under UV light irradiation. The results showed good photodegradation efficiencies, between 92% (ZnS obtained with Na_2_S) and 96% (ZnS obtained with TAA) after a 240 min exposure of dye solution to UV light [24].

Through the refluxing of Zn(NO_3_)_2_·6H_2_O and TU in ethylene glycol at various temperatures (60–100 °C) for 2–6 h, ZnS NPs with different orientations and sizes (10–30 nm) were synthesized by Phuruangrat and co-workers [56]. It was shown that the crystallinity degree, particle size, and photocatalytic activity of the as-synthesized ZnS NPs are dependent on the reaction time and temperature. As a result, the best photocatalytic degradation of organic dyes MB (96.73%) and MO (94.68%) after 120 min of UV light irradiation was reported for ZnS NPs obtained at 100 °C for 6 h, which have a higher degree of crystallinity (crystallite size of 39.45 nm) and larger particle sizes (about 30 nm).

The influence of particles’ morphology on ZnS NSs adsorption–photocatalytic performances were recently investigated [38]. Using ZnO as templates, 1D ZnS NSs, such as nanosheets (Figure 1b), nanotubes (Figure 1c), and short nanotubes, were synthesized by substitution reaction. The average thickness and width of the ZnS nanosheets were ~50 nm and 1 μm. According to TEM and HRTEM analyses, the diameter, shell thickness, and lengths of ZnS nanotubes and short nanotubes were as follows: 100–900 nm and ~300 nm, 30–50 nm and ~30 nm, and 0.5–10 μm and ~1.5 μm. The band gap energy of the nanosheets (3.41 eV, Figure 1b), nanotubes (3.48 eV, Figure 1c), and short nanotubes (3.47 eV) was found to have lower values than that of the bulk ZnS (3.72 eV). This decrease was attributed to the localized electronic levels which could form in the forbidden band, as effect of defects, local bond distortions, and interfaces from ZnS crystalline structure. Based on the adsorption–photocatalysis experiments’ results, ZnS short nanotubes with a porous structure proved better adsorption for MB (10 mg/L), about 25% after 120 min, while ZnS nanosheets showed higher photocatalytic efficiency in MB degradation (96%) after 300 min in UV light (300 W lamp) exposure. The higher exposure ratio of the (1 0 0) crystal planes on nanosheets, compared to that on nanotubes or short nanotubes, has, as effect, not only band gap narrowing but also more active site formation for reactants’ adsorption and electrons/holes trapping [38].

**Figure 4:**
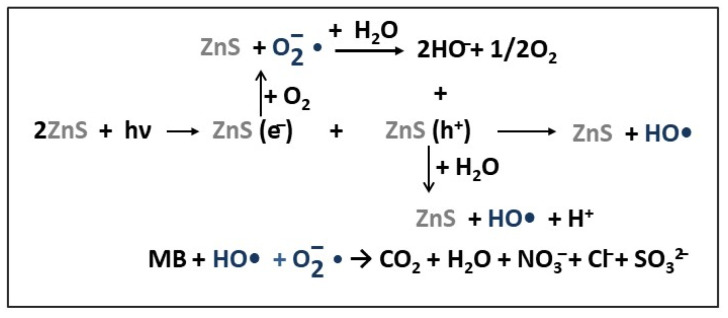
The reaction mechanism proposed for MB dye degradation under UV light irradiation, in presence of 1D ZnS photocatalyst.

Based on the above studies, the mechanism proposed for MB dye photodegradation using 1D ZnS NS catalysts is schematically presented in Figure 4.

Under UV radiation, on the VB and CB of ZnS photocatalyst, photo-induced electron-hole pairs are generated and transferred to the ZnS NPs’ surface. Subsequently, the photogenerated electrons (e^−^) and holes (h^+^) react with dissolved oxygen and water molecules adsorbed on the surface of the ZnS NPs, resulting superoxide anion radicals (O_2_^–^•) and hydroxyl radicals (HO•). These highly reactive species oxidize and decompose MB dye molecules to CO_2_, H_2_O, and other salt ions [56,65].

The effect of capping agents and Ag-doping on the morphology, optoelectronic, and photocatalytic properties of ZnS NS photocatalysts has been the subject of many studies. In recent works [44,49,50], the photocatalytic activity of ZnS and Ag-ZnS catalysts for different dyes’ degradation were studied comparatively, under irradiation with UV light and sunlight, respectively.

Photo-responsive ZnS NSs (ZnSC1 and ZnSC2) were prepared in similar conditions, using MPA and PVP as capping agents, via a simple reflux method followed by calcination [49]. Uncapped ZnS NPs (ZnSUC) were also prepared by the same procedure but without addition of any capping agent. Based on the morphological analysis, ZnS obtained with capping agents showed specific 1D structures consisting of nanorods (ZnSC1) and nanospheres (ZnSC2), while randomly arranged particles were formed in ZnSUC (Figure 1d). This demonstrates that capping agents play a significant role in tailoring ZnS NPs morphology, thus influencing their photocatalytic response.

**Figure 5:**
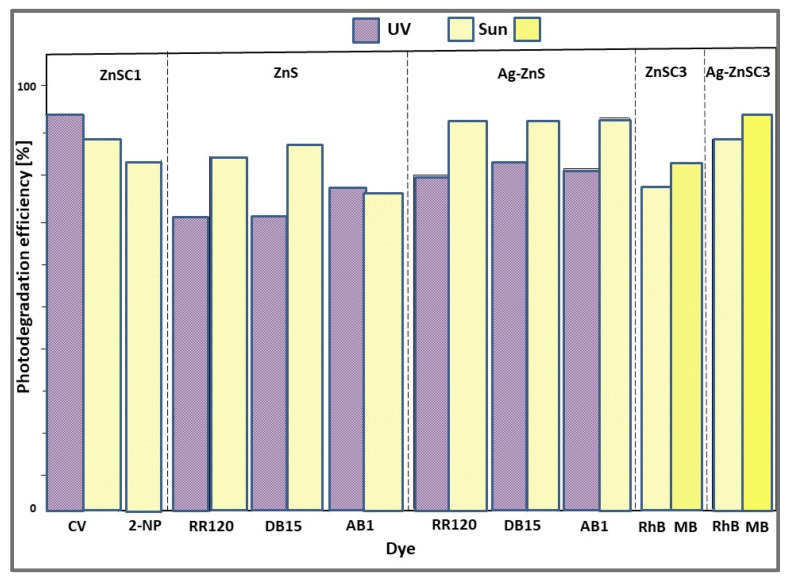
The photocatalytic activity of different ZnS NSs in dye degradation under UV and solar irradiation.

The band gap of 3.89 eV (compared with 3.5 eV for uncapped ZnS), the higher surface area of 252 m^2^/g (five times higher than that of uncapped ZnS), together with the elongated 1D nanorod structure (Figure 1e), which favors the transport of photogenerated charged carriers, and the effective charge separation (according to PL studies) contributed to the enhanced photocatalytic activity of the ZnSC1 catalyst (Figure 5). Thus, capped ZnS nanostructure ZnSC1 degraded 93% of CV dye solution (10 mg/L) under UV light (Hg arc, 125 W, ~1 W/m^2^) after 3 h, while only 54% of CV was decomposed in the presence of uncapped ZnS catalyst in the same photocatalytic conditions. Under sunlight (~782 W/m^2^ and ~40 °C) exposure for 3 h, even if the efficiency of ZnSC1 photocatalyst was slightly decreased to 88%, it was still three times higher than that of ZnSUC (28%), due to the strong interactions of ZnS with the capping agent, and the vacancy levels occurred between energy levels which favor the dye degradation in Vis region [49]. The photocatalytic activity of ZnSC1 catalyst (2 g/L), investigated in the degradation of NP (2-nitrophenol) pollutant (stable, water-soluble, and very toxic, even at low concentrations) under solar irradiation for 3 h showed an efficiency of 83%, as seen in Figure 5.

Furthermore, the capped ZnS NSs proved a good reusability capacity (e.g., the efficiency decreased by 14% for ZnSC1) after three cycles of CV dye photodegradation, thus demonstrating their extended photostability and beneficial impact for environment.

By a simple hydrothermal method using ascorbic acid as reducing agent, ZnS and Ag-doped ZnS (Ag-ZnS with 1% wt Ag) nanostructures were prepared by Ravikumar et al. [50]. The addition of Ag in the ZnS lattice had, as a result, a decrease of crystallite sizes, from 14.3 nm in ZnS to 12.4 mm in Ag-ZnS, due to the Ag^+^ ions diffusion at the ZnS surface, preventing interfacial grain growth (the symmetry-breaking effect). According to FE-SEM and TEM analysis, ZnS NSs contain both spherical and hexagonal-shaped particles, but after doping with Ag^+^, the ZnS morphology became spherical and ball-like, formed by individual nano-sized, round-shaped particles’ aggregation.

The dye (RR120, DB15, and AB1) removal efficiencies of ZnS and Ag-ZnS photocatalysts after UV and sunlight irradiation for 120 min are showed in Figure 5. The best photodegradation efficiencies, evaluated under UV light and sunlight irradiation, were the following:

- 77% for AB1 and 87% for DB15 dyes using ZnS catalyst;
- 83% for AB1 and 94% for DB15 dyes using ZnS-Ag catalyst.

The addition of Ag^+^ in the ZnS structure generates an interstitial energy band between CB and VB which acts as a trapping barrier, restricting the fast electron-hole recombination, thus enhancing the photocatalytic activity of the Ag-doped ZnS. Moreover, the increased photocatalytic performance of the Ag-ZnS photocatalyst is the combined result of the band gap energy (3.11 eV), particle size (12.4 mm), and shape (spherical, ball-like NPs), which facilitates light absorption on the entire region, both in UV and especially in Vis.

The reusability studies showed that Ag-ZnS photocatalyst had high photostability and degraded more than 80% of RR120 under sunlight irradiation, after the fourth cycle [50].

A facile one-step hydrothermal method was also used for the preparation of ZnS (noted ZnSC3) and Ag-doped ZnS (Ag-ZnSC3) with 1D nanospheres structures, as photocatalysts for dye (RhB and MB) degradation under sunlight illumination [44]. In the synthesis procedure, glucose was used both as a capping agent and as a mild reducing agent, due to its special properties such as biodegradability, natural abundance, and low cost. The addition of Ag^+^ in the cubic crystal structure of ZnSC3 does not generate significant changes regarding the crystallite sizes, nanosphere sizes, and band gap energy values. Compared with pristine ZnSC3 NSs, the decrease of crystallite size (from 9.97 nm to 9.28 nm), of nanosphere size (showing a better NPs distribution), and of band gap energy (from 3.88 eV to 3.79 eV) was reported. As a result, the Ag-ZnSC3 catalyst showed enhanced photocatalytic efficiency for the degradation of RhB (86%) and MB (93%) dyes after 120 min of solar irradiation, as is depicted in Figure 5. The enhanced dye photodegradation is attributed to the higher electron transfer in the Ag-doped ZnSC3 catalyst than that in the undoped ZnSC3 catalyst [44].

**Figure 6:**
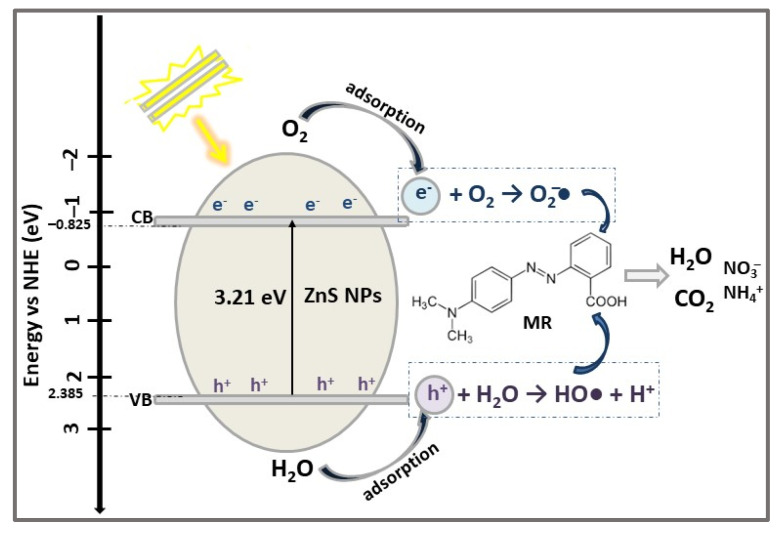
The photocatalytic degradation mechanism of MR dye using ZnS NP photocatalyst in Vis light irradiation for 2 h.

Cubic phase ZnS NPs with an average particle size of about 40 nm were obtained by a simple and facile precipitation method [53]. Optical and PL experiments’ results revealed that ZnS NPs band gap energy is 3.21 eV (suitable for Vis light absorption), and the emission spectra (λ_excit_ = 290 nm) showed four emission bands at 345, 408, 444, and 510 nm, attributed to the surface defects (S vacancies and Zn vacancies/interstitials) on the ZnS NPs surface. The photocatalytic activity of ZnS NPs was studied in the degradation of four azo dyes, two containing −N=N−bonds (MO and MR) and the other two not (MB, XO), under Vis light irradiation (λ = 550 nm, 18 W) for 120 min. The ZnS NPs photodegradation efficiencies for MB, XO, MO, and MR together with their molecular formula and structures are shown in Table 2. The higher photocatalytic degradation of MO and MR dyes is a consequence of the dye structure and photocatalytic mechanism (Figure 6), the active species HO• oxidizing first −N=N−, and the other bonds from dye molecule [53].

More recently, Zhang and co-workers [19] prepared ZnS NSs with a lyotropic liquid crystal template method. The obtained ZnS NSs belong to the cubic sphalerite crystalline phase and are formed of irregular particles with a certain agglomeration and diameters varying from 60 nm to 200 nm. 

Compared with the template-free ZnS catalyst, the ZnS NSs showed more than ten times greater specific surface area (48.08 m^2^/g) and narrower band gap energy (3.23 eV compared with 3.71 eV), resulting in improved absorption of Vis light that favors the separation of photogenerated electron-hole pairs, thus increasing photocatalytic efficiency. The photocatalytic experiments were carried out using 0.02 g ZnS NS photocatalyst, MB as-simulated pollutant (50 mL, 20 mg/L), and a xenon lamp (CELHXF300, 300 W, 20 mW/cm^2^, 350 nm < λ < 780 nm) as Vis light source. The photodegradation efficiency of the ZnS NS photocatalyst was 99.76% after 150 min of illumination, while for template-free ZnS it was 90.95%. Based on reusability tests, after four cycles of MB degradation, photocatalyst efficiency decreased slightly from 99.76% to 96.03%, due to its incomplete recovery. 

### 2.3. 3D ZnS-Based Nanostructures

The 3D ZnS urchin-like nanoparticles (ZnS ULNPs) were prepared by a simple, template free, and one-step hydrothermal method, using zinc acetate dihydrate, TSC, and EDA as precursors [37]. In the 3D NSs, ZnS ULNPs of the hexagonal wurtzite phase are formed by a large number of microspheres particles, with an average size of 60 nm, on the surface, of which nanorods are perpendicularly grown, resulting a stable 3D structure (particles with low energy). This specific 3D structure is built from nanorods formed in the hydrothermal process, based on the liquid–solid transformation. According to UV–Vis spectroscopy and N_2_ adsorption–desorption isotherm analysis, mean pore diameter (13.8 nm), total pore volume (0.0728 m^3^/g), porosity (about 90% belonging to mesopores), band gap energy (3.84 eV), and BET surface area (20.8 m^2^/g) were evaluated.

The photocatalytic activity and potential application in wastewater treatment of ZnS ULNPs (70 mg) were investigated in MO dye solution (100 mL, 3.75 ppm) degradation at pH = 5.5 under Vis and UV light irradiation. The results showed that 3D ZnS ULNPs have no effect on MO solution photodegradation in Vis light, while an efficiency of 80% was obtained when the system was exposed in UV light for 60 min. Although the photodegradation efficiency of MO (one of the most persistent organic pollutants) is not very high compared to that of 1D ZnS NSs (e.g., 94.68% in 120 min, [56]), 3D ZnS NSs can be considered promising photocatalysts for organic pollutant removal from wastewater.

## 3. Zinc Sulfide-Based Heterostructures Photocatalysts for Organic Pollutant Degradation

Besides morphology-tailoring and doping with metal ions, topics already covered in this review, other key strategies for enhancing ZnS NSs photocatalytic efficiency using the band gap engineering are the control of intrinsic defects’ (sulfur and zinc vacancies) amount and the heterostructures (composites) construction of two or more components (inorganic/organic semiconductors, carbon-based materials, etc.). Although doping and surface control improve light (UV, Vis, solar) absorption, the rapid recombination of photo-induced electrons and holes in a bare ZnS semiconductor still remain an open issue for further research. The development of ZnS-based heterojunction structures offers at least two advantages, such as the photo-response range broadening (from UV into Vis region) and the photogenerated charge carriers’ separation efficiency increasing [65].

Based on our knowledge, there are few published reviews on bare ZnS and ZnS-based heterostructures’ development (synthesis, properties) and prospective applications [16,18,66]. If the synthesis and photocatalytic activity of ZnS-based nanocomposites, with CQDs, C nanotubes, GO/RGO were reported extensively in other review article [66], in this part of the paper are discussed approaches related to recent (last three years) ZnS-based heterostructures (mainly with metal oxides/sulfides) developed as photocatalysts for organic pollutant degradation in wastewater and issues that were not considered in the previously mentioned publications.

### 3.1. ZnS-Based Photocatalysts with n-n Heterojunction Structure

Tungsten trioxide (WO_3_) is an n-type semiconductor with band gap in the range 2.6–3 eV, high-charge carrier mobility (excellent conductivity), and (photo)corrosion stability in a wide pH range [39,67]. However, the application of bare WO_3_ as a photocatalyst is limited due to its lower CB energy level, which favors the rapid recombination of e^−^/h^+^ pairs and slows down the O_2_ reduction to superoxide radical anions (O_2_^−^•), with effect on organic pollutant degradation. As for bare ZnS, it is also a n-type semiconductor characterized by a fast photogeneration of e^−^/h^+^ pairs and a CB with a highly negative reduction potential.

Using a simple co-precipitation method, Murillo-Sierra et al. [33] obtained the coupled photocatalysts ZnS/WO_3_ by varying the ZnS concentration (1, 5, and 10 wt%). For comparison, bare WO_3_ and ZnS semiconductors were separately prepared by precipitation, using Na_2_WO_4_∙9H_2_O, Zn(NO_3_)_2_∙2H_2_O, and Na_2_S∙9H_2_O as precursors, respectively.

In the ZnS/WO_3_ heterostructure photocatalysts containing ZnS cubic phase and γ-WO_3_ monoclinic phase, ZnS NPs were deposited on the WO_3_ surgace, without crystalline lattice doping. The surface morphology analysis of ZnS/WO_3_ composite showed that small flakes of ZnS NPs were uniformly distributed on the surface of WO_3_ particles with sheet-like shape morphology. Band gap energy values for ZnS/WO_3_ photocatalyst are in the range 2.61–2.64 eV, very close to that of WO_3_ (2.6 eV) and significantly lower than ZnS band gap energy (3.5 eV). These values are in accordance with crystallite size (e.g., 2.52 nm for ZnS compared to 29.5 nm for ZnS/WO_3_ with 10 wt% ZnS and Eg = 2.64 eV) and specific surface area (e.g., 91.18 m^2^/g for ZnS and 20.67 m^2^/g for ZnS/WO_3_ with 10 wt% ZnS) variations. The increase of surface area by about 25%, when ZnS is added to WO_3_ (S_BET_ = 16.34 m^2^/g), was attributed to the high surface area of ZnS and its fast dispersion onto the WO_3_ surface.

**Figure 7:**
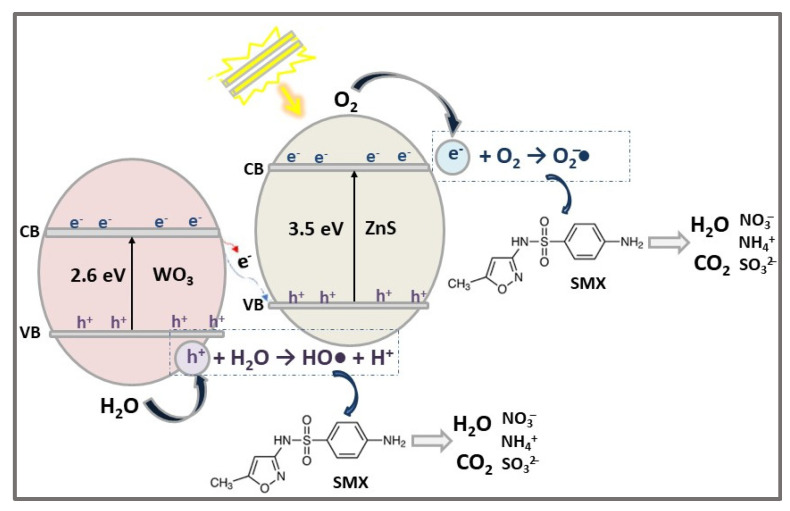
The photocatalytic degradation mechanism of SMX antibiotic using ZnS/WO_3_ heterostructure photocatalyst under Vis light irradiation for 1 h.

The photocatalytic degradation of the highly recalcitrant SMX antibiotic (20 mg/L) under simulated sunlight (Sunset XLS + solar simulator) irradiation was investigated for coupled ZnS/WO_3_ photocatalysts (0.5 g/L pollutant solution) together with single ZnS and WO_3_ semiconductors. After 60 min of illumination, the photodegradation of SMX was almost complete (99%) in the presence of ZnS/WO_3_ heterostructure photocatalyst with 5 wt% ZnS, compared with 38.5% for WO_3_, and about 55% for ZnS. This enhanced photocatalytic activity of ZnS/WO_3_ composite was attributed to the combined effects of higher specific surface area, high particle dispersion, suitable band gap energy, and reduced charge carriers recombination rate (confirmed by PL analysis) [33]. The proposed SMX photodegradation mechanism by ZnS/WO_3_ heterostructure photocatalyst is illustrated in Figure 7.

### 3.2. ZnS-Based Photocatalysts with n-p Heterojunction Structure

Among metal oxides, which generally are n-type (wide band gap) semiconductors, e.g., SnO_2_ (Eg = 3.4 eV [68]), TiO_2_ (Eg = 3.25 eV [69]), and ZnO (Eg = 3.1–3.39 eV [70,71]), Ag_2_O and CuO are p-type semiconductors. Silver oxide (Ag_2_O) is a narrow band gap semiconductor with a band gap of about 1.2 eV (enhanced light absorption in the visible region), cubic crystal structure, and excellent electronic transport properties [72]. Cupric oxide (CuO) is an inexpensive and non-toxic narrow band gap semiconductor material (Eg = 1.2 eV–1.9 eV) with a monoclinic crystal structure [73]. Therefore, a heterojunction structure, formed by n-type semiconductor ZnS with a p-type semiconductor (Ag_2_O, CuO), is expected to improve the photo-response of pristine ZnS in Vis region. 

In this context, the n-p heterojunction structure ZnS/Ag_2_O, prepared by a simple chemical precipitation method, was characterized and tested as a Vis-light-driven photocatalyst for a model organic pollutant MB degradation [45]. For comparison, ZnS and Ag_2_O semiconductors were separately obtained by hydrothermal and precipitation methods. The ZnS/Ag_2_O photocatalyst is composed of hexagonal wurtzite ZnS and cubic phase Ag_2_O and, according to SEM images, Ag_2_O NPs (d = 200–300 nm) were uniformly covered on the rough surface of ZnS broccoli-like microspheres, with an average size around 3–4 μm. 

The band gap energy values for the ZnS, Ag_2_O, and ZnS/Ag_2_O composite were found to be 3.52 eV, 1.80 eV, and 2.85 eV, respectively, confirming the significant reduction of ZnS band gap energy in the heterostructure formed with Ag_2_O. The photocatalytic activities of ZnS, Ag_2_O, and ZnS/Ag_2_O (0.05 g/100 mL dye solution) were evaluated in the degradation of MB dye (10 mg/L), under illumination with a 300 W Xe lamp for 50 min. The results showed that ZnS/Ag_2_O photocatalyst degraded 92.4% of MB, almost three times and one and four tenths times more than ZnS and Ag_2_O, respectively. The recycling experiments demonstrated that the ZnS/Ag_2_O heterostructure photocatalyst remains active even after three cycles of MB degradation [45].

**Figure 8:**
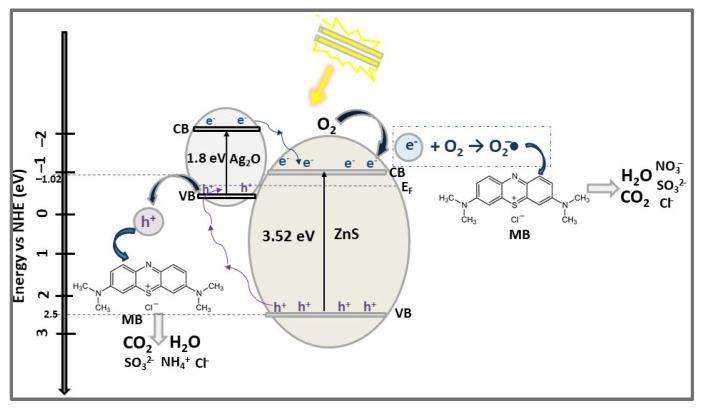
The photocatalytic degradation mechanism of MB dye using ZnS/Ag_2_O heterostructure photocatalyst under Vis light irradiation for 50 min.

Based on the results obtained from the experiments of MB photocatalytic degradation by the ZnS/Ag_2_O composite, alone and with different radical scavengers, the possible mechanism presented in Figure 8 was proposed [45]. Before the ZnS/Ag_2_O composite construction, the Fermi levels (E_f_, dashed line in Figure 8) of n-type semiconductor ZnS and p-type semiconductor had different potentials. With the formation of the ZnS/Ag_2_O composite and its irradiation by Vis light, the photogenerated electrons are transferred from ZnS (with higher CB potential) to Ag_2_O until the quasi-Fermi level (quasi-static equilibrium) is generated. As a consequence, the CB and VB energy bands of ZnS are shifted down, and those of Ag_2_O are shifted up, with equalization of the two components’ Fermi levels. Therefore, driven by the potential difference, the photogenerated electrons from Ag_2_O CB are transferred to ZnS CB and reduce the surface chemisorbed O_2_ to oxidizing radicals O_2_^−^•, which degrade MB into small molecules. Meanwhile, the holes on the ZnS VB are transferred to Ag_2_O VB and oxidize MB dye directly. Due to the narrower band gap, Ag_2_O significantly contributes to the enhancement of the photocatalytic activity of the ZnS-based heterostructure catalyst by extending the photo-response into the Vis region, thus enhancing the photon efficiency, and, as a result, the generation and transfer of charge carriers are more efficient.

Recently, the ZnS–CuO/PVA/Chitosan nanohybrid photocatalyst, consisted of spherical CuO NPs loaded on a ZnS nanoflower supported on carbon framework PVA/Chitosan, was obtained using co-precipitation and an ultrasonic-assisted procedure [57]. Chitosan and PVA are known as supportive carbon matrices for metal sulfide and oxide nanoparticles, providing more active sites for the pollutant photodegradation and, therefore, the enhancement of the nanocomposites’ performance. The abundance of zinc vacancies (identified by EPR and FTIR measurements), not only in surface but also in the subsurface of ZnS, favors two-photon excitation that improves Vis-light harvesting. Accordingly, the ZnS–CuO/PVA/Chitosan nanohybrid band gap energy of 2 eV is significantly lower compared with that of ZnS (3.65 eV) and slightly higher than that of CuO (1.7 eV). The more than two times higher surface area of the ZnS–CuO/PVA/Chitosan photocatalyst (106.147 m^2^/g), compared with CuO and ZnS, is due to the large number of CuO NPs distributed or accumulated on the ZnS surface, which could provide more active sites for photo-oxidation/reduction reactions.

As a representative of low-biodegradable pharmaceutical compounds having high hydrophilicity and low volatility, tetracycline (TC) is an antibiotic with a wide antibacterial spectrum and low cost widely used as a bacteriostatic agent for treating infections [74,75]. Therefore, the treatment of wastewater containing TC is absolutely necessary. For this purpose, the photocatalytic activity of ZnS–CuO/PVA/Chitosan catalyst (50 mg/100 mL TC solution) was tested in degradation of TC (25 mg/L) under Vis light (800 W tungsten halogen lamp) illumination for 180 min. The enhanced synergetic effect of ZnS–CuO/PVA/Chitosan was confirmed by its photocatalytic efficiency of 94.7%, obviously higher compared to that of ZnS (40%) and CuO (60%). Also, the ZnS-based heterostructure photocatalyst showed to have high stability in that the TC degradation of 94.7% (1st cycle) remained at 93.5% after six cycles [57].

The photocatalytic mechanism was elucidated using scavenger tests, and both oxidizing species O_2_^−^• and HO• were found to play key roles in TC degradation. 

As efficient metal-oxide-based photocatalysts, ferrites are widely used in the formation of heterojunction structures with other semiconductors. Spinel ferrite CuFe_2_O_4_ is an environmentally friendly p-type semiconductor with a narrow band gap (Eg = 1.32–1.69 eV), good magnetic properties, high chemical stability, and low cost [76].

The novel n-p heterojunction nanocomposite ZnS/CuFe_2_O_4_ was prepared via two-step chemical co-precipitation for the removal of organic dyes (MB and CV) under Vis light irradiation [59]. According to FESEM analysis, ZnS/CuFe_2_O_4_ nanocomposite showed a flake-like morphology, with individual particle sizes of 50-100 nm. 

**Figure 9:**
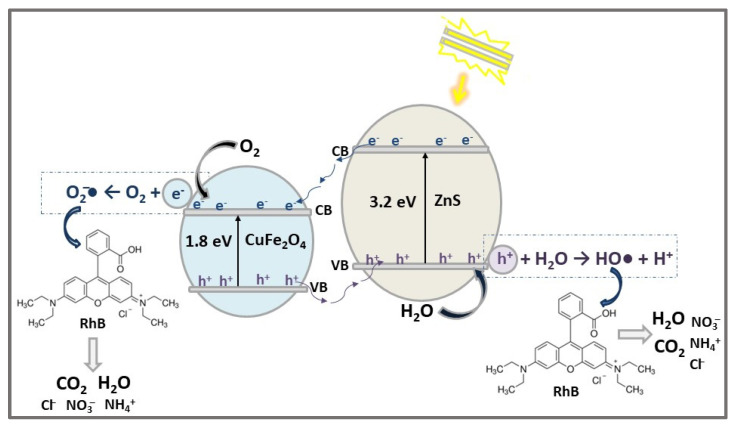
The photocatalytic degradation mechanism of RhB dye using ZnS/CuFe_2_O_4_ heterostructure photocatalyst under Vis light irradiation for 2 h.

The band gap energies of ZnS, CuFe_2_O_4_, and ZnS/CuFe_2_O_4_ were found to be 3.2 eV, 1.8 eV, and 2.19 eV, respectively. Compared with ZnS, the optimal absorption of the ZnS/CuFe_2_O_4_ nanocomposite was shifted to the visible light wavelengths; as a result, the photocatalytic activity in the Vis light range was improved. To explain the photocatalytic activity of ZnS/CuFe_2_O_4_, 1 mg of photocatalyst was used for the degradation of 100 mL MB/CV solution (30 mg/L initial concentration) exposed for 2 h to Vis light produced by a 200 W tungsten halogen lamp. The obtained results highlighted that degradation efficiency of MB and CV dyes was higher in the presence of ZnS/CuFe_2_O_4_ composite (82% and 87%), while only 47% MB and 50% CV were degraded when ZnS catalyst was used. This difference could be explained by the dye photodegradation mechanism illustrated in Figure 9. The photogenerated electrons (e^−^) from ZnS CB are transferred to CuFe_2_O_4_, due to the highly negative reduction potential of ZnS compared to that of CuFe_2_O_4_, and then interact with adsorbed O_2_ molecules to form superoxide anions radicals (O_2_^−^•) which oxidate dye molecules. In the meantime, photogenerated holes (h^+^) on CuFe_2_O_4_ transferred to ZnS VB react with H_2_O molecules to produce strongly active oxidizers hydroxyl radicals (HO•), which also decompose dye molecules (e.g., RhB) in CO_2_, H_2_O, and other ions.

According to reusability experiments, it was reported that the ZnS/CuFe_2_O_4_ nanocomposite demonstrates superior photocatalytic performance through up to four cycles of dye degradation in Vis light exposure [59].

Ag_8_SnS_6_ (ATS) is a narrow band gap (Eg = 1.24–1.41 eV) semiconductor used in photoelectrochemical salt-water splitting thermoelectric devices, as light absorbers in solar cells, and as a photocatalyst in dye degradation [77]. In order to investigate the MB dye degradation under Vis light irradiation, Wang and co-workers [58] prepared the ZnS/ATS heterostructure photocatalyst by heating ATS (obtained by a stepwise heating method), zinc acetate, and thiourea in ethylene glycol. The ATS NPs, with irregular polygon morphology and crystal size varying from 35 to 120 nm, were transformed to cotton-shape particles with the addition of spherical-like ZnS (sphalerite cubic phase) NPs during the synthesis procedure. Based on Tauc-plot analysis, the optical band gaps were estimated at 3.85 eV, 1.15 eV, and 1.5 eV, for ZnS, ATS, and ZnS/ATS nanocomposite. The photocatalytic activity of ATS, ZnS, and ZnS/ATS (150 mg/100 mL dye solution) was tested in degradation of MB dye solution (5 mg/L), under illumination with a metal halide lamp (400 W) for 5 h. Without any catalyst, the photodegradation of MB solution was about 40%, and increased to 50.4%, 65.8%, and 85.5%, in the presence of ZnS, ATS, and ZnS/ATS (0.5 mmol in ATS) catalysts. As reported, the photocatalytic performance of ZnS/ATS heterostructure photocatalyst is mainly due to the increased separation and transfer efficiency of photogenerated electron-hole pairs at ZnS/ATS grain boundaries [58].

### 3.3. ZnS-Based Photocatalysts with Z-Scheme Heterojunction Structure

GaOOH is a wide band gap semiconductor (Eg = 4.4 eV) and promising material for UV transparent conductors and solar-blind photodetectors, due to its optical transparency in the visible and near UV region [78]. Coupled with a narrow band semiconductor (e.g., ZnS), GaOOH forms heterojunction structures with photocatalytic activity in organic pollutant removal from wastewater. Hence, the photocatalytic efficiency of unprecedented ZnS/GaOOH heterojunction structures, prepared via a one-pot hydrothermal process by varying the reaction time from 1h to 9h, was investigated in the degradation of dyes (e.g., RhB) and antibiotics (TC, CIP, LF) under Vis light irradiation [20]. 

SEM and TEM analysis showed that the heterojunctions are formed by ZnS spheres gradually attaching to the GaOOH nanorods as the hydrothermal synthesis time increases. However, when the hydrothermal time exceeds 3 h, the ZnS spheres become oversized, resulting in mismatched heterojunctions. 

The Eg values of ZnS and GaOOH, estimated as 3.48 eV and 4.49 eV, are in accordance, or close, to those reported in the literature [38,49,78]. The photocatalytic experiments’ results showed that the ZnS/GaOOH heterojunction photocatalyst achieved higher efficiency (86.4%) in TC removal after 150 min in Vis light irradiation compared with that of individual components, ZnS (60.4%) and GaOOH (61.9%). Lower degradation efficiencies were obtained for CIP (30%) and LF (20.3%) antibiotics after 60 min in Vis light irradiation in the presence of heterojunction catalysts. Photocatalytic tests for RhB dye degradation, in similar conditions, demonstrated that ZnS/GaOOH composites are performant photocatalysts, degrading ~90% of RhB in 5 min.

**Figure 10:**
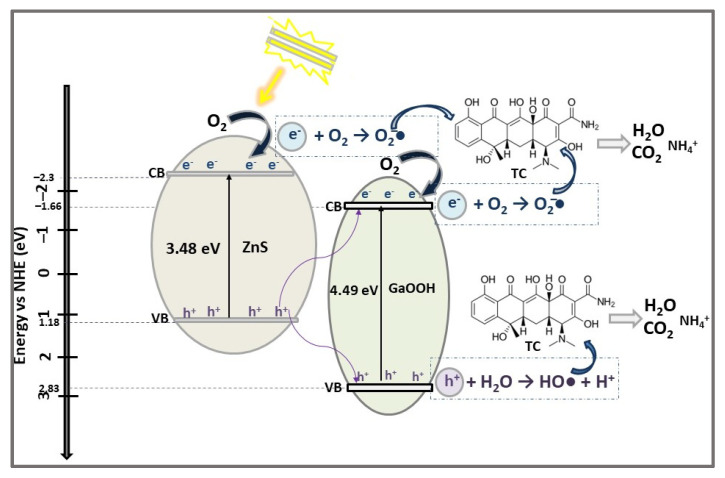
The photocatalytic degradation mechanism of TC antibiotic using ZnS/ GaOOH heterostructure photocatalyst under Vis light irradiation for 150 min.

To elucidate the TC photocatalytic degradation mechanism by ZnS/GaOOH composite, the photocatalytic redox active species (O_2_^−^•, HO•, h^+^ and e^−^) were investigated by using different sacrificial reagents, such as AA, TBA, EG, and Cr_2_O_3_. As seen in Figure 10, the electrons from ZnS CB and GaOOH CB can reduce O_2_ to O_2_^−^• active radicals due to their CB potentials which are more negative than that of O_2_/O_2_^−^• (−0.33 eV). The holes on the GaOOH VB transform OH^−^ ions, adsorbed on the photocatalyst surface from the TC solution, to HO• because the GaOOH VB (2.83 eV) potential is higher than 1.89 eV for OH^−^/HO• [79]. Accordingly, the enhanced photocatalytic performance is the result of the efficient charge carriers’ separation and transport, and spatially separated reduction and oxidation sites in ZnS/GaOOH heterojunction, which act as Z-scheme heterojunction photocatalyst (confirmed by photodeposition and the ESR experiments’ results).

## 4. Conclusions

During the past decades, various ZnS and ZnS-based photocatalysts with applications in wastewater/air treatment, CO_2_ reduction, and H_2_ production via photoelectrolytic water splitting were reported. 

**Figure 11:**
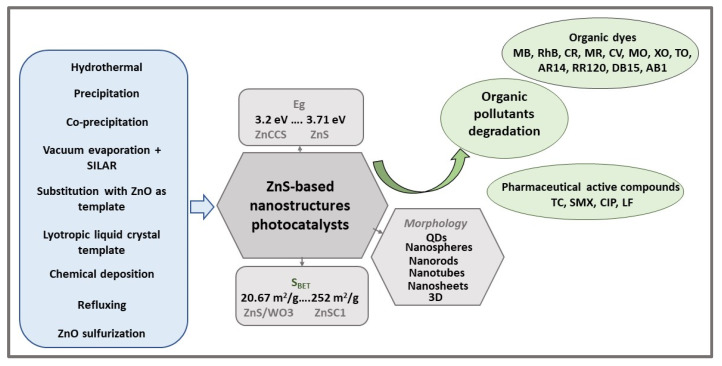
Summary of synthesis methods, specific properties, and applications in wastewater treatment of ZnS-based nanostructure photocatalysts.

This literature review, which highlights the recent developments of ZnS-based photocatalysts for wastewater treatment, has shown that, in a relatively short time (five years), considerable efforts have been made related to this topic, thus providing valuable information for the research community in this field. A summary of the review is systematically presented in Figure 11.

To improve the ZnS photocatalytic activity in the Vis light range, various strategies, such as morphology engineering, metal doping, and heterojunctions construction, have been used to date. In this regard, a particular importance should be devoted to the design and development of stable ZnS-based heterostructures to enhance the photocatalytic performance of a single ZnS photocatalyst, prone to photo-corrosion, and to extend the photocatalytic response in the Vis region.

Furthermore, the various morphologies of the ZnS-based photocatalyst, tailored by varying synthesis parameters, have a strong influence on its photocatalytic performance, affecting the distribution of photogenerated electron-hole pairs and the adsorption ability for reactant molecules. Therefore, the techniques used for ZnS-based photocatalyst preparation must be facile, low-cost, and energy-efficient with the possibility to industrial-scale application; green techniques are more preferred as easy and low-cost ways to remove from the wastewater both organic pollutants and plant wastes.

Not least, the ZnS-based photocatalysts’ reusability and photostability studies are necessary to evaluate their ability to regenerate several times (more than four cycles) without a significant loss in organic pollutant photodegradation efficiency.

The photocatalytic performances of ZnS nanostructures and ZnS-based heterostructures in the removal of organic pollutants from industrial wastewater may represent important starting/comparison data for the further development of various ZnS-based photocatalysts with improved solar energy conversion efficiency for environmental remediation and green energy production.

## Figures and Tables

**Table 1:** ZnS-based photocatalysts for organic pollutants degradation in wastewater treatment.

Photocatalyst	Structure	Synthesis Method	Pollutant Conc. (mg/L)	Catalyst Dosage (g/L)	Light Source	η* (%)	t (min)	Ref.
ZnS Au-ZnS Ag-ZnS	0D NsS QDs	HT	MB (30)	0.05	UV (6 W lamp)	72.5 96.7 92.6	180	[48]
ZnS Mn-ZnS	0D NsS QDs	Refluxing	TO (12.65)	0.5 g	UV (8 W lamp)	92.6 94	30 90	[3]
ZnS Gd-ZnS	0D NsS QDs	Refluxing	AR14 (20)	0.2	UV	85.4 91.1	180	[51]
ZnCCSs	ZnS QDs	HT	CR (50)	0.2	UV (350 W Hg lamp)	95.94	120	[6]
ZnS	0D NsS QDs	PP at mild temperatures (60–80 °C)	MB (2.6)	0.1	VIS (1000 W halogen lamp)	61	180	[55]
ZnS	1D NSs nanospheres	Capping free HT	MB (12)	0.1	UV	96	240	[24]
ZnS	1D NSs nanospheres	Refluxing	MB (3.2) MO (3.27)	1	UV (three black light lamps)	96.73 94.68	120	[56]
ZnS	1D NSs nanosheets short nanotubes nanotubes	Substitution reaction using ZnO as templates	MB (10)	0.2	UV (30 W lamp)	96 92 88	300	[38]
ZnS	1D NSs	Sulfuration of ZnO NPs obtained by PP	PNP (10)	1.5	UV (12 W/m^2^ LEDs)	51	240	[47]
ZnS	1D NSs nanorods nanospheres	Refluxing using different capping agents + calcination	CV (10.2) 2-NP(10.2)	2	UV (Hg arc, 125 W) Solar Solar	54–93 28–88 83	180	[49]
ZnS Ag-ZnS	1D NSs spherical balls	HT	RR120 (50) DB15 (50) AB1 (50)	0.1	UV-A Solar UV-A Solar	71–77 75–87 80–83 94	120	[50]
ZnS Ag-ZnS	1D NSs nanospheres	HT	RhB (2.4) MB (1.6)	1	Solar	77 86 84 93	120	[44]
ZnS	1D NSs ZnS NPs	Lyotropic liquid crystal template	MB (20)	0.4	VIS light (300 W Xe lamp)	99.76	150	[19]
ZnS	1D NSs ZnS NPs	PP	MB (50) XO (50) MO (50) MR (50)	0.05	VIS (18 W lamp)	78.41 81.22 90.90 95.10	120	[53]
ZnS	1D NSs ZnS NPs	PP	CR (30)	0.2	Vis (45 W halogen lamp)	98.78	90	[5]
ZnS	3D NSS ZnS urchin-like particles	HT	MO (3.75)	0.7	UV-C (Philips lamps)	80	60	[37]
ZnS/TiO2	HNSs n-n heterojunction	Chemical deposition	AB113 (25)	0.185	UV-A (400 W Kr UV lamp)	99.2	27	[30]
ZnS/WO_3_	HNSs n-n heterojunction	CoPP	SMX (20)	0.5	VIS (Sunset XLS + solar simulator)	95	60	[33]
ZnS/Ag_2_O	HNSs n-p heterojunction	PP	MB (10)	0.5	VIS (300 W Xe lamp)	92.4	50	[45]
ZnS-CuO/PVA/Chit	HNSs ZnS-CuO n-p heterojunction	CoPP	TC (25)	0.5	VIS (800 W tungsten halogen lamp)	94.7	180	[57]
ZnS/ATS	HNSs n-p heterojunction	One step heating	MB (5)	1.5	VIS (400 W metal halide lamp)	85.5	300	[58]
ZnS/CuS/pC	HNSs n-p heterojunction	Vacuum evaporation+ SILAR	MB (20)	0.5	Vis (300W UV lamp)	98	60	[11]
ZnS/CuFe_2_O_4_	HNSs n-p heterojunction	CoPP	MB (30) CV (30)	0.01	VIS (200 W tungsten halogen light)	82 87	120	[59]
ZnS/GaOOH	HNSs Z-scheme heterojunction	HT	TC (20) CIP (20) LF (20) RhB (5)	0.4 0.2	VIS light (300 W Xe lamp)	86.4 31.6 20.3 89.8	150 60 5	[20]

η* is pollutant degradation efficiency after t min of irradiation.

**Table 2:** The structural, optical, and photocatalytic properties of undoped and metal-doped ZnS QDS’ photocatalysts.

Photocatalyst	Eg (eV)	Particle Size (nm)	Photodegradation				
			Dye		η* (%)	Time (min)	Ref.
			Chemical Formula and Structure	λ_max_ (nm)			
ZnS QDs	4	4.2	Methylene Blue, MB C_16_H_18_N_3_ClS 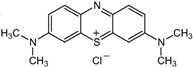	665	61 (Vis)	180	[55]
ZnS QDs	4.64	4.3			72.5	180	[48]
Ag-ZnS QDs	3.06	4.7			92.6		
Au-ZnS QDs	2.4	5.6			96.7		
ZnS QDs	3.7	21–44	Acid red 14, AR14 C_20_H_12_N_2_Na_2_O_7_S_2_ 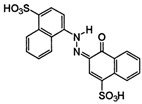	515	85.4	180	[51]
Gd-ZnS QDs	3.45	20–34			91.1		
ZnS QDs	3.7	~4	Tropaeolin O, TO C_12_H_9_N_2_NaO_5_S 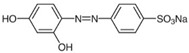	426	92.6	30	[3]
Mn-ZnS QDs	3.7	~4			94	90	
ZnCCSs	3.55	15	Congo Red, CR C_32_H_22_N_6_Na_2_O_6_S_2_ 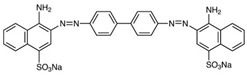	510	95.94	120	[6]
ZnS NPs	3.21	40	Methylene Blue, MB C_16_H_18_N_3_ClS 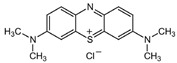	665	78.4 (Vis)	120	[53]
			Xylenol orange, XO C_31_H_32_N_2_O_13_S 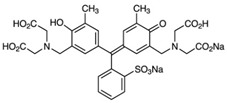	583	81.2 (Vis)		
			Methyl Orange, MO C_14_H_14_N_3_NaO_3_S 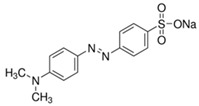	463	90.9 (Vis)		
			Methyl Red (acid red 2), MR C_15_H_15_N_3_O_2_ 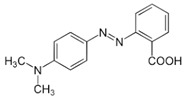	425	95.1 (Vis)		

η* is pollutant degradation efficiency after t min of irradiation.

## Abbreviations

QDs—quantum dots; NCs—nanocomposites; HT—hydrothermal; CBD—chemical bath deposition; PP—precipitation; CoPP—Co-precipitation; SILAR—successive ionic layer adsorption and reaction; ESR—electron spin-resonance; AR14—acid red 14 Y; TO—Tropaeolin O; CR—Congo red; CV—crystal violet; RR120—reactive red 120; DB15—direct blue 15; AB1—acid black 1; XO—Xylenol orange; MR—methyl red; AB113—acid blue 113; PNP—para-nitrophenol; SMX—sulfamethoxazole; CIP—ciprofloxacin; LF—levofloxacin; TAA—thioacetamide; TSC—thiosemicarbazide; EDA—ethylenediamine; PVA—polyvinyl alcohol; MPA—3-mercaptopropionic acid; PVP—polyvinylpyrrolidone; CCSs—cellulose/chitosan sponge; TSH—para-toluene sulfonyl hydrazide; pC—porous carbon; AA—ascorbic acid; BAB—tertbutyl alcohol; EG—ethylene glycol; CB—conduction band; VB—valence band.
